# Comparative evaluation of four mosquitoes sampling methods in rice irrigation schemes of lower Moshi, northern Tanzania

**DOI:** 10.1186/1475-2875-8-149

**Published:** 2009-07-06

**Authors:** Eliningaya J Kweka, Aneth M Mahande

**Affiliations:** 1 Tropical Pesticides Research Institute, Division of livestock and human disease vector control, P.O. Box 3024, Arusha-Tanzania; 2 Tropical Pesticides Research Institute, Mabogini field station, Moshi, Tanzania

## Abstract

**Background:**

Adult malaria vector sampling is the most important parameter for setting up an intervention and understanding disease dynamics in malaria endemic areas. The intervention will ideally be species-specific according to sampling output. It was the objective of this study to evaluate four sampling techniques, namely human landing catch, pit shelter, indoor resting collection and odour-baited entry trap.

**Methodology:**

These four sampling methods were evaluated simultaneously for thirty days during October 2008, a season of low mosquitoes density and malaria transmission. These trapping methods were performed in one village for maximizing homogeneity in mosquito density. The cattle and man used in odour-baited entry trap were rotated between the chambers to avoid bias.

**Results:**

A total of 3,074 mosquitoes were collected. Among these 1,780 (57.9%) were *Anopheles arabiensis *and 1,294 (42.1%) were *Culex quinquefasciatus*. Each trap sampled different number of mosquitoes, Indoor resting collection collected 335 (10.9%), Odour-baited entry trap-cow 1,404 (45.7%), Odour-baited entry trap-human 378 (12.3%), Pit shelter 562 (18.3%) and HLC 395 (12.8%). General linear model univariate analysis method was used, position of the trapping method had no effect on mosquito density catch (DF = 4, F = 35.596, P = 0.78). Days variation had no effect on the collected density too (DF = 29, F = 4.789, P = 0.09). The sampling techniques had significant impact on the caught mosquito densities (DF = 4, F = 34.636, P < 0.0001). The Wilcoxon pair-wise comparison between mosquitoes collected in human landing catch and pit shelter was significant (Z = -3.849, P < 0.0001), human landing catch versus Indoor resting collection was not significant (Z = -0.502, P = 0.615), human landing catch versus odour-baited entry trap-man was significant (Z = -2.687, P = 0.007), human landing catch versus odour-baited entry trap-cow was significant (Z = -3.127, P = 0.002).

**Conclusion:**

Odour-baited traps with different baits and pit shelter have shown high productivity in collecting higher densities of mosquitoes than human landing catch. These abilities are the possibilities of replacing the human landing catch practices for sampling malaria vectors in areas with *An. arabiensis *as malaria vectors. Further evaluations of these sampling methods need to be investigated is other areas with different species.

## Background

Human landing catch (HLC) practices have been used for a long time in estimating the human malaria vectors abundance. It has been a most reliable method in estimating abundance because it targets the host-seeking behaviour of mosquitoes [1,2]. The entomological inoculation rate and vectorial capacity are important indices of malaria transmission. A main component of both entomological inoculation rate and vectorial capacity is the human biting rate (i.e. the number of female anopheline mosquitoes biting a person per night). The main negative impact underlying this traditional method of sampling mosquitoes is the variation of attractiveness between individuals [3,4], as well as ethical and practical reasons for minimizing the use of this method [5]. Due to these negative impacts of the HLC, an alternative sampling method has to be found and used in its place. The method should be considered to be similarly or more sensitive than HLC, preferably it should be useable in different conditions than HLC, and it should be acceptable in protecting human participation in disease vector sampling. Apart from HLC, other sampling methods were evaluated in this study, including odour-baited entry trap (OBET), indoor resting collection (IRC) and pit shelter (PS). The odour-baited trap was formerly used to evaluate the mosquito responsiveness and preference to host odours [6,7]. Indoor resting collection method has been used before for the estimation of the indoor resting density, as described in WHO manual [1]. Pit shelters have been used for the estimation of the outdoor resting mosquito density [1]. All these sampling methods where done individually in different sites, they have shown to be efficient in sampling the malaria vectors.

Therefore, it was the objective of this study to compare the performance of human landing catch against odour-baited entry trap, Indoor resting collection and pit shelters in sampling (trapping) malaria vectors, within the same environmental setting in lower Moshi rice irrigation schemes in northern Tanzania.

## Methods

### Study area description

The study area was in Mabogini village within the lower Moshi rice irrigation schemes. The detailed description of the study area is given in Ijumba *et al *[8] and Kweka *et al *[9].

### Adult sampling

The sampling of mosquitoes was done during a four-week period in October, 2008. The four sampling methods evaluated were operated in the same time. Human landing catch (HLC), odour-baited trap (OBET), pit shelters (PS) and Indoor resting collection (IRC). IRC was performed in the morning from 6:30 am to 8:30 am of every experimental day in cowshed and indoors using mechanical aspirator as described in entomological manual book [1]. Pit shelters were sampled every morning from 7:00 am to 7:30 am, the pit dimensions were as described in entomology manual [1] for collecting outdoor resting mosquitoes density. The OBET was used as described by Costantini *et al *[10]. The trap is composed of a tent with a either a man or cow whose odours are drawn to a cage trap by a fan via polythene tunnel. OBET dimensions were height 2 meters, length 2 meters and width 1.5 meters. For HLC, the same man (Tanzanian, 34 years old) exposed his feet while using mechanical aspirator for collecting landing mosquitoes at each collection site, one collector worked from 6:00 p.m. to 6:00 a.m, mosquitoes were sorted by an hour interval. OBET used both man and cow: the mosquitoes seeking for hosts were collected in a protected chamber before reaching the host.

### Field identification of mosquitoes

The mosquitoes in OBET, indoor resting collection and pit shelters were collected from each sampling method with a mechanical aspirator in the morning into well labeled paper cups for identification. Anopheline mosquitoes were sorted and identified morphologically to species according to the keys of Gillies and De Meillon [11]. For each collection method, an abdominal condition of each mosquito was identified as described in entomological manual book [1].

### Ethical issues

Before the study implementations, villagers were called for a meeting to be given information about the aim of the study. After the meeting, people participating in HLC, OBET, pit shelter and indoor resting collection-catch were chosen and invited for consent. They were given written consent forms, as all were literates. Signing of consent form was witnessed for each participant by a non-study member. They were screened for malaria parasites every Friday and medication was available free-of-charge. Fortunately, none was found parasite positive during the study period.

### Statistics

Data entry and validation were done in ms-excel 2003 version. Data analyses were performed using the SPSS version 15.0 for windows. In analysing the variables, which are influencing mosquito collection, such as days, sampling method (trap) and position, were performed using the generalized linear model univariate analysis. The comparison between HLC and other sampling methods (i.e. HLC versus OBET-man, HLC versus OBET-cow and HLC versus Indoor resting collection in cowshed) were computed using non-parametric test of two related-samples tests (Wilcoxon pair-wise comparison). The abdominal conditions (unfed, fed, semi gravid and gravid) of collected mosquitoes were expressed in percentages in all trapping techniques used.

## Results

**Figure 1:**
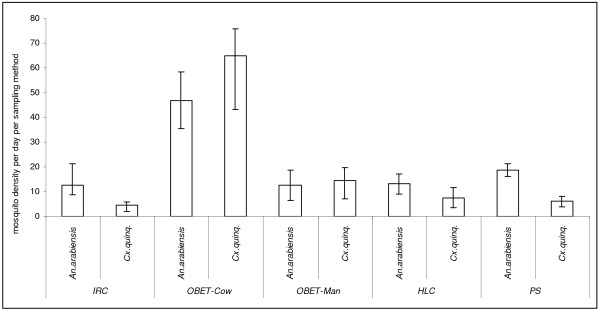
**Mosquitoes abundance caught by each evaluated sampling method**.

**Figure 2:**
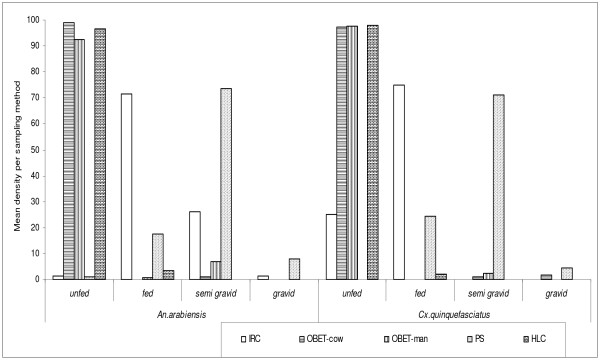
**The abdominal conditions of *Anopheles arabiensis *and *Culex quinquefasciatus *caught in each sampling method**.

A total of 3,074 mosquitoes was collected. Among these 1,780 (57.9%) were *Anopheles arabiensis *and 1294 (42.1%) were *Culex quinquefasciatus*. Each method sampled different number of mosquitoes, indoor resting collection collected 335 (10.9%), OBET-cow 1,404 (45.7%), OBET-man 378 (12.3%), Pit shelter 562 (18.3%) and HLC 395 (12.8%). The density distribution results are shown in Figure 1. The abdominal status of mosquitoes collected is shown in Figure 2.

In the evaluation of these sampling methods, a total of 150 (30 days) experiments were done. In these experiments, OBET trap with human or cow were treated as two different traps due to different hosts occupied traps. Therefore the total number of sampling methods was five. General linear model univariate analysis method was used, position of the sampling method had no effect on mosquito density catch (DF = 4, F = 35.596, P = 0.78). Days variation had no effect on the collected density too (DF = 29, F = 4.789, P = 0.09). The sampling methods had significant impact on the caught mosquito densities (DF = 4, F = 34.636, P < 0.0001). Further analysis was done to compare between the individual sampling method against HLC. The Wilcoxon pair wise comparison between mosquitoes collected in HLC and Pit shelters was significant (Z = -3.849, P < 0.0001), HLC versus indoor resting collection was not significant (Z = -0.502, P = 0.615), HLC versus OBET-human was significant (Z = -2.687, P = 0.007), HLC versus OBET-cow was significant (Z = -3.127, P = 0.002).

Species identification was not done as the previous studies in the area showed that more than 95% of the *Anopheles gambiae *sensu lato were *An. arabiensis *[12].

## Discussion

The results of this study have demonstrated the usefulness of using different methods for sampling disease vectors in surveillance and control programmes in disease endemic areas for the purpose of developing better option than human landing catch. The field evaluation of HLC, PS, OBET-human, OBET-cow and IRC in the same ecological setting enabled the efficient comparison of these sampling methods in mosquito collection. In this study, only IRC had no statistical significant results in sampling mosquitoes against HLC (Z = -0.502, P = 0.615), The main advantage of PS is that, it samples the outdoor-resting mosquitoes, while IRC samples the indoor resting mosquitoes but in both mosquitoes have different abdominal status [1]. OBET-human, OBET-cow and HLC had advantages much as they were sampling host-seeking mosquitoes [7,10,12].

OBET-cow caught the highest number of anopheline followed by the PS, the least was IRC preceded by OBET-human and HLC (Figure 1 and 2). These results are supported by the previous OBET trap studies [7,13], and reports contrary to what found in Madagascar by Laganier *et al *[14] using the OBET trap. The high proportion of mosquitoes caught in PS method were gravid, semi-gravid and fed, which reflects the number of mosquitoes resting while seeking for oviposition sites and blood meal digestion [1]. In this context, HLC seems to be weaker and more risk to the collector than other sampling methods where volunteer is protected from host-seeking mosquitoes. This is supported by the other study compared HLC and odour-baited resting boxes in the same study areas [9]. In this study, the higher number of mosquitoes collected in OBET-cow, PS and with IRC in cowshed might have been a result of the exophilic and zoophilic behaviour of the vectors in this area [7-9,11].

In this study factors, such as position of sampling methods, days and time, did not appear to influence mosquito collection. Only the sampling method used seemed to have influenced the number of vectors collected, but different sampling methods have shown varying ability in collecting mosquitoes of different abdominal condition, which can be more informative in malaria epidemiology than only have unfed host-seeking vectors in HLC.

In vector population currently, there is a high level of insecticide resistance against malaria vectors [15]. This might influence the high transmission rate despite of the coverage of the ITNs. These findings are merely supported by other findings reported by Rubio-Palis and Curtis [5].

There is a need to advocate other methods for sampling mosquitoes other than HLC in areas with *An. arabiensis *for surveillance and control purposes, as other potential sampling methods are available [1,9,13]. In OBET-human and OBET-cow, both visual and physical stimuli are present. Host-seeking mosquitoes are first attracted by the olfactory stimulus and then will move toward the host via additional host stimuli such as visual cues, temperature, and humidity [3,16,17]. Thus, the mosquito in response to such stimuli can target the appropriate site for taking its blood meal before coming in contact with human or cow.

Despite the differences observed in the mean numbers of vectors collected in this study, the age population structure of the malaria vectors were overall similar in HLC and OBET with cow or human, while it was similar for PS and IRC. These odour-baited based methods have provided qualitatively comparable abilities in collecting host-seeking malaria vectors populations as found previously for HLC and light traps [12,17-20].

Due to the increase of malaria drug resistance distribution [21] and more needs of measuring the disease exposure OBET with human or cow, and PS offers a reasonable alternative to HLC, because they do not expose the collector to malaria infected mosquitoes and because collection can be done overnight without interruptions. Based on the findings of this study, OBET with human or cow could be used to explain the malaria transmission estimation and exposure better than HLC [12,13].

## Conclusion

The use of host-seeking sampling techniques should be considered for further evaluation in different ecological settings. This will be more appropriate in planning for the campaigning for ceasing human landing catch practices. The ecological characteristics should be used to deploy the appropriate sampling technique for existing vectors.

## Competing interests

The authors declare that they have no competing interests.

## Authors' contributions

EJK and AMM contributed equally to this study, in conceiving, its design, collecting and analysing data, and in writing this manuscript. Both agreed with the final version before submission.
